# Nonlinear relationship between aspartate aminotransferase to alanine aminotransferase ratio and the risk of prediabetes: A retrospective study based on chinese adults

**DOI:** 10.3389/fendo.2022.1041616

**Published:** 2022-10-25

**Authors:** Changchun Cao, Xiaohua Zhang, Junhu Yuan, Yibing Zan, Xin Zhang, Chao Xue, Yulong Wang, Xiaodan Zheng

**Affiliations:** ^1^ Department of Rehabilitation, Shenzhen Dapeng New District Nan’ao People’s Hospital, Shenzhen, Guangdong, China; ^2^ Department of Orthopedics, Foshan First People’s Hospital, Foshan, Guangdong, China; ^3^ Department of Neurology, Shenzhen Samii International Medical Center (The Fourth People’s Hospital of Shenzhen), Shenzhen, Guangdong, China

**Keywords:** AST/ALT ratio, prediabetes, insulin resistance, nonlinearity, metabolic syndrome

## Abstract

**Objective:**

Recent evidence has revealed that the aspartate aminotransferase to alanine aminotransferase ratio (AST/ALT ratio) may be closely associated with metabolic syndrome and insulin resistance. However, it is unclear whether the AST/ALT ratio correlates with prediabetes risk. The aim of our study was to examine the association between AST/ALT ratios and the risk of prediabetes among a large cohort of Chinese subjects.

**Methods:**

This retrospective cohort study recruited 75204 Chinese adults with normoglycemia at baseline who underwent physical examinations at the Rich Healthcare Group from 2010 to 2016. The AST/ALT ratio at baseline was the target independent variable, and the risk of developing prediabetes during follow-up was the dependent variable. Cox proportional-hazards regression was used to evaluate the independent association between the AST/ALT ratio and prediabetes. This study identified nonlinear relationships by applying a generalized additive model (GAM) and smooth curve fitting. In order to assess the robustness of this study, we performed a series of sensitivity analyses. Moreover, we performed a subgroup analysis to evaluate the consistency of the association in different subgroups. Data from this study have been updated on the DATADRYAD website.

**Results:**

The AST/ALT ratio was negatively and independently related to the prediabetes risk among Chinese adults (HR: 0.76, 95% CI: 0.75-0.84, P<0.0001) after adjusting demographic and biochemical covariates. Furthermore, a nonlinear relationship between the AST/ALT ratio and the risk of developing prediabetes was found at an inflection point of 1.50 for the AST/ALT ratio. When the AST/ALT ratio was to the left of the inflection point (AST/ALT ratio ≤ 1.50), the AST/ALT ratio was negatively related to the prediabetes risk (HR:0.70, 95%CI: 0.65-0.76, P<0.0001). In contrast, the relationship tended to be saturated when the AST/ALT ratio was more than 1.50 (HR: 1.01, 95%CI: 0.89-1.15, P=0.8976). Our findings remained robust across a range of sensitivity analyses. Subgroup analysis revealed that other variables did not alter the relationship between the AST/ALT ratio and prediabetes risk.

**Conclusion:**

This study revealed that AST/ALT ratio was negatively and independently associated with prediabetes risk among Chinese participants. The relationship between the AST/ALT ratio and prediabetes risk was nonlinear, and AST/ALT ratio ≤ 1.50 was strongly inversely correlated with prediabetes risk.

## Background

Diabetes Mellitus (DM) is a metabolic disorder with chronic hyperglycemia. Epidemiological data disclosed by the International Diabetes Federation shows that in 2019, about 463 million people aged 20-79 were found to have diabetes worldwide, with an incidence rate of 9.3% (1). Diabetes is one of the most common metabolic diseases and imposes a heavy economic burden on patients and their countries (2). Prediabetes refers to a pathological stage of blood glucose levels that are in the range between normal levels and diabetic levels. Prediabetes is not only a high-risk factor for diabetes, but also increases the risk of developing chronic kidney disease, cardiovascular disease, and retinopathy (3). In recent epidemiological studies, prediabetes is prevalent in China at approximately 35.7% (4), much higher than other chronic diseases. It has been reported that about 5-10% of people with prediabetes develop DM each year. More than 70% of people with prediabetes eventually develop DM (4). In recent years, patients with impaired glucose tolerance and diabetes have shown a younger trend (5, 6). A poor prognosis is common in young patients with impaired glucose tolerance and diabetes, and they are more likely to develop cardiovascular disease and microvascular complications (7). Moreover, lifestyle interventions could prevent 58% of prediabetic patients from developing DM, according to the Diabetes Prevention Program Research Group (8). Therefore, detection and treatment of prediabetes at an early stage are crucial to preventing DM.

It is well known that nonalcoholic fatty liver disease (NAFLD) and DM often coexist in individuals (9). Recent epidemiological studies have established that subjects with NAFLD are two times more likely to develop DM than normal subjects (10), and more than half of subjects with DM will suffer from NAFLD in the future (11). Alanine aminotransferase (ALT) and aspartate aminotransferase (AST) play a variety of physiological roles and have consistently been recognized as important markers of NAFLD (12). Several studies have reported that the AST to ALT ratio (AST/ALT ratio) accurately identifies insulin resistance (IR) and is considered a potential biomarker (13). Furthermore, some researchers have revealed that the AST/ALT ratio may also be useful for assessing disease risks, such as NAFLD, diabetes mellitus, hyperinsulinemia, cardiovascular disease, and metabolic syndrome (14–17). These findings all indicated the AST/ALT ratio is a good indicator of blood glucose metabolism. However, whether the AST/ALT ratio is related to prediabetes in Chinese adults is unknown. This study aimed to quantitatively examine the specific association between AST/ALT ratio and prediabetes risk, and determine the inflection point of the effect of the AST/ALT ratio on prediabetes in large Chinese subjects.

## Methods

### Data source

We downloaded the raw data freely from the DATADRYAD database (www.datadryad.org) uploaded by Chen et al. (18) from: Association of body mass index and age with the risk of DM in Chinese adults: a population-based cohort study. Dryad Digital Repository. https://datadryad.org/stash/dataset/doi:10.5061%2Fdryad.ft8750v). A total of 685,277 Chinese individuals in multiple regions and cities (Suzhou, Chengdu, Shenzhen, Nanjing, Shanghai, Wuhan, Guangzhou, Beijing, Hefei, Changzhou, and Nantong) were enrolled from 2010 to 2016 for the original study and completed at least a second visits.

### Study population

**Figure 1:**
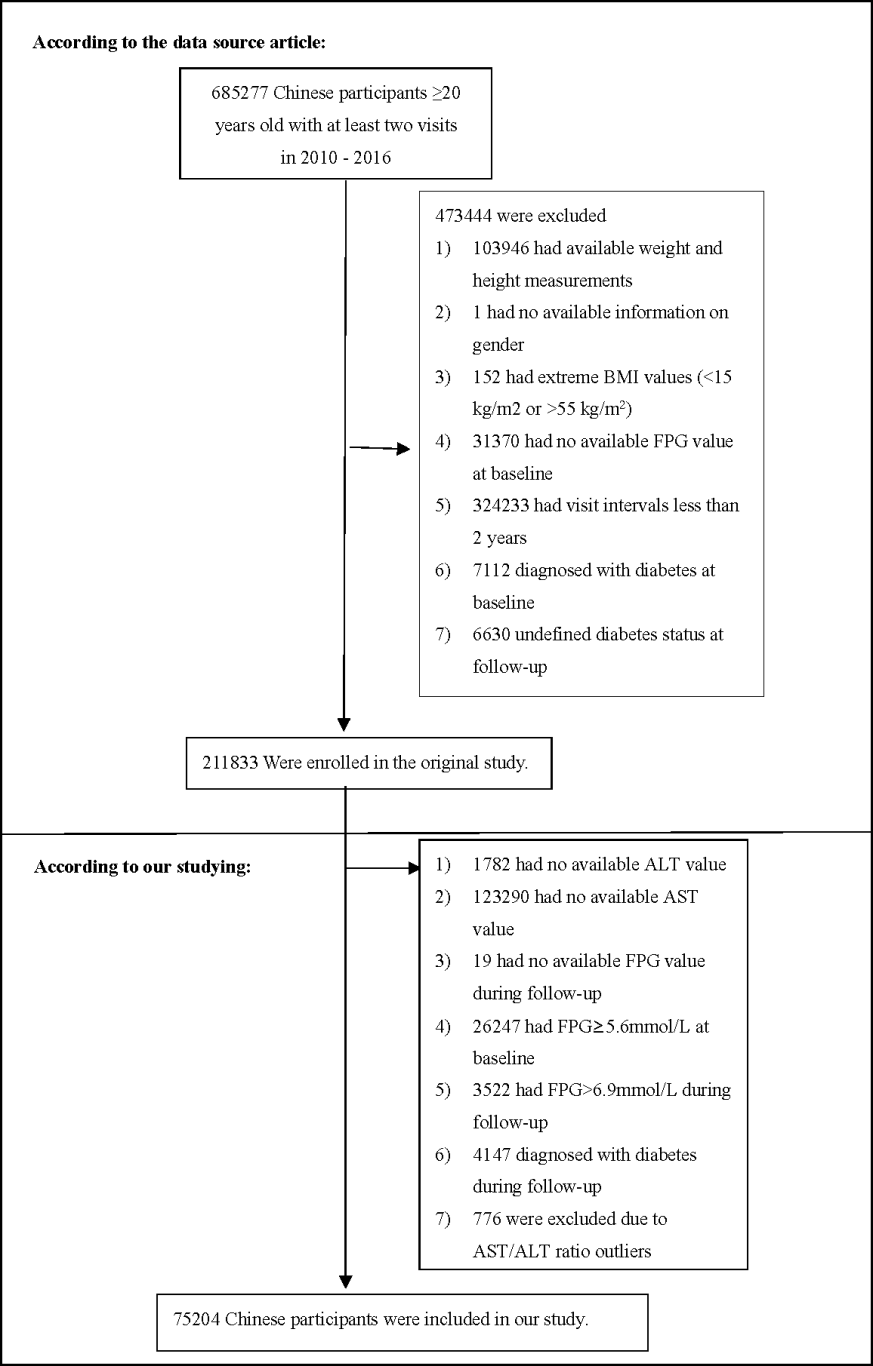
Study Population.

If subjects met any of the following criteria, they were excluded from the study:(1) not defined DM status at follow-up; (2) DM at baseline and follow-up; (3) body mass index (BMI) outliers (BMI>55 or <15 kg/m^2^; (4) missing gender and weight, height, AST, ALT, fasting plasma glucose(FPG) values at baseline, and FPG during follow-up; (5) baseline FPG≥5.6mmol/L and FPG>6.9mmol/L during follow-up; (6) follow-up interval less than two years; (7) those with AST/ALT ratio outliers (out of the range of means plus or minus three standard deviations). Finally, 75204 subjects eventually entered the study. The selection process of the participants was described in detail in **Figure 1**.

### Data collection

All data were collected and collated by trained staff. The initial research obtained the laboratory inspection indicators based on standardized conditions, and treated them following uniform procedures. The trained staff collected demographic variables, such as age, body weight, height, and blood pressure. Participants were measured for height and weight without shoes and light clothing. The BMI was counted by weight (kilograms): the square of height (meters) ratio. Trained staff measure blood pressure by standard mercury sphygmomanometer. And the trained staff used an autoanalyzer (Beckman 5800) to measure clinical variables, including high-density lipoprotein cholesterol (HDL-C), total cholesterol (TC), FPG, low-density lipoprotein cholesterol (LDL-C), triglyceride (TG), AST, ALT, and serum creatinine (Scr), blood urea nitrogen (BUN). The detailed process of calculation of the AST/ALT ratio was described as follows: [serum AST(U/L)]/[serum ALT(U/L)].

### Diagnosis of prediabetes

The diagnosis of prediabetes is based on impaired fasting blood glucose, and the FPG value of patients with prediabetes is between 5.6 and 6.9 mmol/L (19).

### Statistical analysis

We explored the characteristics of all subjects at baseline according to quartiles of the AST/ALT ratio. Skewed and normally distributed continuous variables are described as median (quartile) and mean ± standard deviation, respectively. The differences among the AST/ALT ratio groups were compared by the one-way ANOVA test, the Kruskal-Wallis H test, and the chi-square test. We described the incidence rates in terms of person-year incidence and cumulative incidence (20).

There were 7 (0.01%), 8 (0.01%), 1003 (1.32%), 1003 (1.32%), 33803 (44.49%), 33525 (44.12%), 5592 (7.36%), 2981 (3.92%), 57538 (75.73%), and 57538 (75.73%) subjects with missing data for SBP, DBP, TC, TG, HDL-C, LDL-C, BUN, SCr, drinking status, and smoking status, respectively. To deal with the missing data of covariants, we employed multiple imputations. The imputation model included sex, age, family history of diabetes, drinking status, smoking status, SBP, DBP, BMI, TC, TG, HDL-C, LDL-C, SCr, BUN, and FPG. The processes for missing data analysis made use of the missing at random assumptions (21).

The univariate Cox regression analysis was employed to evaluate the effect of each variable on prediabetes risk. The multivariate Cox regression analysis was also used to explore the exact association of the AST/ALT ratio with the risk of prediabetes. Furthermore, we constructed three models to assess the association between AST/ALT ratio and prediabetes risk: non-adjusted, minimally-adjusted, and fully-adjusted. We only adjusted for these covariances if the matching hazard ratios changed by ≥10% when added to the adjusted model (22).

The present study employed a series of sensitivity analysis to assess that the findings were robust. We converted AST/ALT ratio into a categorical variable according to the quartile and calculated the P for the trend to verify the results of the AST/ALT ratio as the continuous variable and examine the possibility of nonlinearity. Obesity, the elderly and alcohol consumption were associated with an increased risk of prediabetes. Hence, in other sensitivity analyses, we excluded individuals with BMI≥24kg/m^2^, age≥60 years, or drinker (current-drinker and ex-drinker) to assess the relationship between AST/ALT ratio and prediabetes risk. Furthermore, the present study employed a generalized additive model (GAM) to incorporate the continuity variables into the equation as a curve to examine the robustness of our findings. To assess the impact of potential unmeasured confounding factors between the AST/ALT ratio and the risk of prediabetes, we further calculated E-values (23).

Since the AST/ALT ratio was a continuous variable, we tried to identify the nonlinear correlation between the AST/ALT ratio and prediabetes through a smooth curve fitting (penalty curve method) and a GAM. If the relationship was nonlinear, we employed the two-piece linear regression model to determine the inflection point (24). The present study employed the log-likelihood ratio to describe the most appropriate model for the association of the AST/ALT ratio and prediabetes.

Moreover, we applied the Cox proportional hazard model to the subgroup analysis (gender, age, DBP, SBP, BMI, smoking status, drinking status, and family history of diabetes). According to median or clinical cut point (25), age (<60, ≥60 years), BMI (<24, ≥24 kg/m2), SBP(<140, ≥140mmHg), and DBP(<90, ≥90mmHg) were converted into categorical variables. And each stratification has undergone a fully adjusted analysis, except for the stratification factor. To examine subgroup interactions, likelihood ratio tests were performed (26, 27). Comparisons of survival rates and cumulative event rates were carried out using the Kaplan-Meier method. In addition, we compared the Kaplan-Meier hazard ratios (HR) of adverse events by log-rank test (28). STROBE statement was followed for all results (22, 29).

Statistical analysis was employed using the R software package (www.r-project.org, The R Foundation) and Empower-Stats (www.empowerstats.com, X&Y Solutions, Inc., Boston, MA). Statistical significance was determined by P < 0.05 in two-tailed tests.

## Results

### Characteristics of individuals

**Table 1** presented all eligible individuals’ basic clinical measurements, biochemical tests, and other parameters. The final analysis included 75204 individuals, with a mean age of 40.90 ± 12.09 years and a female participation rate of 44.27%. The average BMI was 23.03 ± 3.25 kg/m2, the average AST/ALT ratio was 1.24± 0.45, and the average FPG was 4.78± 0.49 mmol/L. The incidence of prediabetes was 13.08% (9839/75204). According to quartiles of the AST/ALT ratio, individuals were created into four groups (≤0.90, 0.90-1.20, 1.20-1.53, and >1.53). As a result of the study, it was found that the individuals in the bottom AST/ALT group had higher DBP, SBP, BMI, TG, LDL-C, TC, ALT, Scr, lower age, and low HDL-C. In addition, a higher proportion of men and smokers and a lower proportion of never drinker in the bottom AST/ALT ratio group.

**Table 1:** The baseline characteristics of participants .

AST/ALT ratio	Q1(≤0.90)	Q2(0.90 to ≤1.20)	Q3(1.20 to ≤1.53)	Q4(>1.53)	P-value
**Participants**	18791	18811	18725	18877	
**Gender**					<0.001
** Male**	16020 (85.25%)	12189 (64.80%)	8353 (44.61%)	5347 (28.33%)	
** Female**	2771 (14.75%)	6622 (35.20%)	10372 (55.39%)	13530 (71.67%)	
**Age(years)**	39.16 ± 9.81	41.77 ± 11.85	41.84 ± 12.80	40.83 ± 13.39	<0.001
**Drinking status**					
** Current-drinker**	486 (2.59%)	434 (2.31%)	380 (2.03%)	279 (1.48%)	
** Ex-drinker**	4263 (22.69%)	3189 (16.95%)	2264 (12.09%)	1591 (8.43%)	
** Never- drinker**	14042 (74.73%)	15188 (80.74%)	16081 (85.88%)	17007 (90.09%)	
**Smoking status**					<0.001
** Current-smoker**	4572 (24.33%)	3465 (18.42%)	2306 (12.32%)	1445 (7.65%)	
** Ex-smoker**	1163 (6.19%)	850 (4.52%)	552 (2.95%)	353 (1.87%)	
** Never-smoker**	13056 (69.48%)	14496 (77.06%)	15867 (84.74%)	17079 (90.48%)	
**Family history of diabetes**					0.622
** No**	18406 (97.95%)	18455 (98.11%)	18369 (98.10%)	18520 (98.11%)	
** Yes**	385 (2.05%)	356 (1.89%)	356 (1.90%)	357 (1.89%)	
**SBP (mmHg)**	122.36 ± 14.96	119.14 ± 15.67	116.51 ± 15.81	114.23 ± 15.97	<0.001
**DBP (mmHg)**	76.69 ± 10.70	74.24 ± 10.56	72.30 ± 10.29	70.85 ± 10.06	<0.001
**BMI (kg/m2)**	25.13 ± 3.19	23.40 ± 3.00	22.25 ± 2.86	21.35 ± 2.65	<0.001
**AST/ALT ratio**	0.71 ± 0.13	1.05 ± 0.09	1.35 ± 0.10	1.85 ± 0.27	<0.001
**HDL-C (mmol/L)**	1.29 ± 0.29	1.35 ± 0.29	1.40 ± 0.29	1.44 ± 0.29	<0.001
**TG (mmol/L)**	1.41 (0.99-2.10)	1.10 (0.78-1.60)	0.90 (0.66-1.30)	0.80 (0.60-1.11)	<0.001
**LDL-C (mmol/L)**	2.81 ± 0.68	2.71 ± 0.66	2.64 ± 0.66	2.59 ± 0.65	
**TC (mmol/L)**	4.81 ± 0.90	4.67 ± 0.88	4.59 ± 0.87	4.53 ± 0.87	<0.001
**BUN(mmol/L)**	4.84 ± 1.16	4.73 ± 1.18	4.59 ± 1.19	4.41 ± 1.17	<0.001
**SCr(umol/L)**	76.64 ± 13.87	72.69 ± 16.01	68.45 ± 15.51	65.57 ± 15.98	
**FPG (mmol/L)**	4.82 ± 0.48	4.80 ± 0.48	4.76 ± 0.49	4.73 ± 0.50	<0.001

Values are n (%) or mean ± SD.

AST/ALT, aspartate aminotransferase to alanine aminotransferase; SBP, systolic blood pressures; DBP, diastolic blood pressures; BMI, body mass index; ALT, alanine aminotransferase; AST aspartate aminotransferase; HDL-C, high-density lipoprotein cholesterol;, LDL-C, low-density lipoprotein cholesterol; TC, total cholesterol; TG, triglycerides; Scr, serum creatinine; BUN, blood urea nitrogen; FPG, fasting plasma glucose.

### The incidence rate of prediabetes

9839 individuals developed incident prediabetes during follow-up, as shown in **Table 2**. The total prevalence rate of all individuals was 13.08% (12.84%–13.32%). Specifically, the prevalence rates of the four AST/ALT ratio groups were 17.06% (16.52%–17.60%), 13.88% (13.39%–14.37%), 11.91% (11.45%–12.37%), and 9.49% (9.07%–9.91%), respectively. In the overall population, the morbidity rate was 4149.62 per 100,000 person-years. Specifically, the morbidity rate of the four AST/ALT ratio groups were 5436.46 per 100,000 person-years, 4387.93per 100,000 person-years, 3757.58 per 100,000 person-years, and 3022.78 per 100,000 person-years, respectively. Individuals with higher AST/ALT ratios had a lower cumulative prevalence and incidence of prediabetes than those with lower AST/ALT ratios (p<0.001 for trend).

**Table 2:** Incidence rate of incident prediabetes.

AST/ALT ratio	Participants (n)	prediabetes events (n)	Cumulative incidence (95% CI)(%)	Per 100,000 person-year
Total	75204	9839	13.08 (12.84–13.32)	4149.62
≤0.90	18791	3206	17.06(16.52–17.60)	5436.46
0.90-1.20	18811	2611	13.88 (13.39–14.37)	4387.93
1.20-1.53	18725	2230	11.91 (11.45–12.37)	3757.58
>1.53	18877	1792	9.49 (9.07–9.91)	3022.78
P for trend			<0.001	0.001

**Figure 2:**
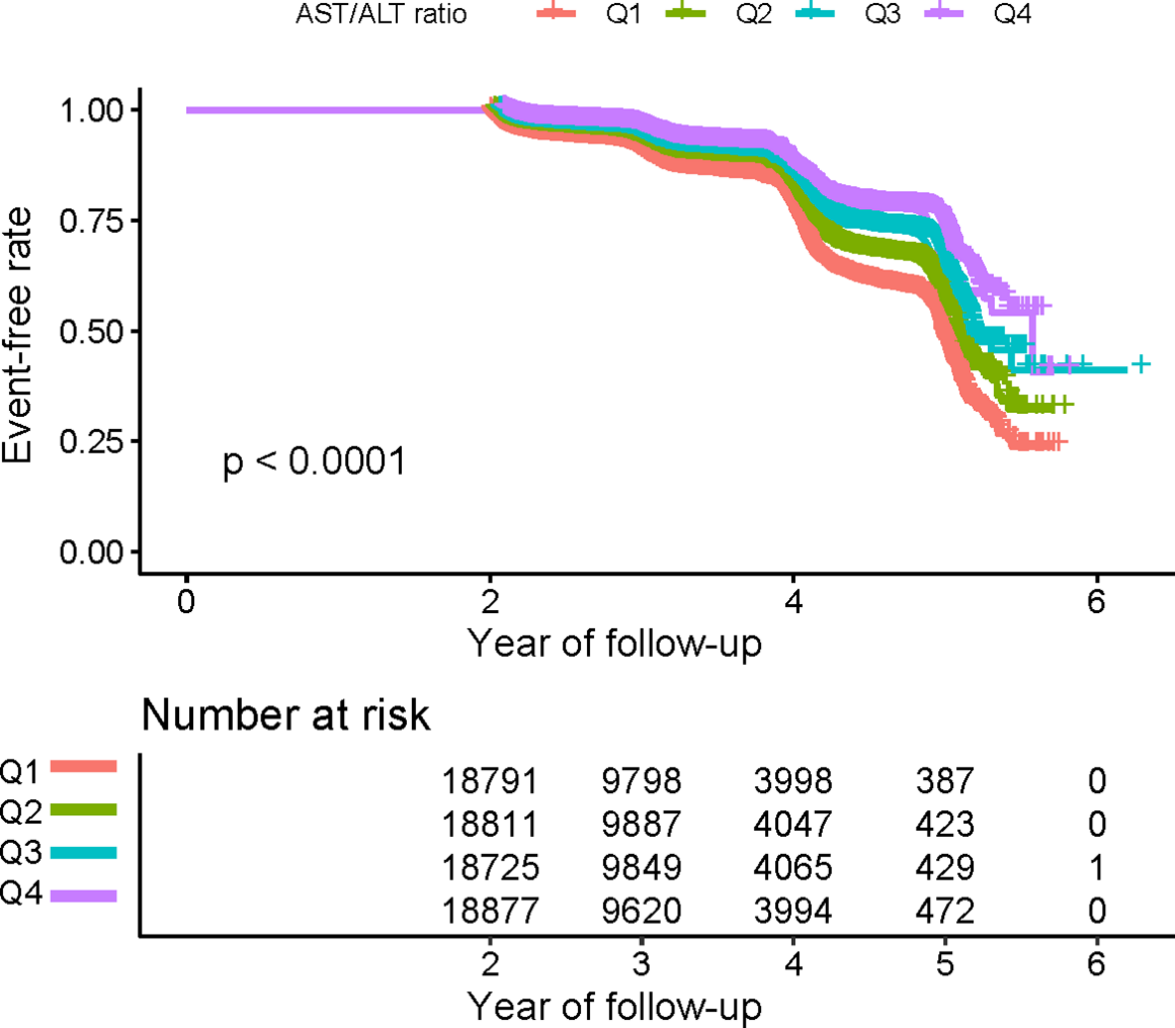
Kaplan–Meier event-free survival curve. Kaplan–Meier event-free survival curve. Kaplan–Meier analysis of incident prediabetes based on AST/ALT ratio quartiles (log-rank, P < 0.0001).

Kaplan-Meier curves for the probability of prediabetes-free survival are depicted in **Figure 2**. A significant difference existed between the four AST/ALT groups in terms of prediabetes risk (P < 0.0001). As AST/ALT levels increased, there was a gradual increase in the probability of prediabetes-free survival. Therefore, individuals in the top AST/ALT group had the lowest risk of prediabetes.

### Univariate analysis

The univariate analysis demonstrated the following results in **Table 3**. There was a positive association between sex, age, SBP, DBP, BMI, TC, TG, LDL-C, SCr, BUN, FPG, and the risk of prediabetes. In contrast, HDL-C and the AST/ALT ratio are inversely associated with prediabetes risk. Meanwhile, never drinker and never smoker have a lower risk of prediabetes. A higher risk of prediabetes was found in males compared to females.

**Table 3:** The results of univariate analysis.

	Statistics	HR (95% CI)	P value
**Gender**			<0.0001
**Male**	41909 (55.73%)	ref	
** Female**	33295 (44.27%)	0.66 (0.64, 0.69)	
** Age(years)**	40.90 ± 12.09	1.04 (1.04, 1.04)	<0.0001
**Drinking status**			
** Current-drinker**	1579 (2.10%)	ref	
** Ex- drinker**	11307 (15.04%)	0.65 (0.58, 0.72)	<0.0001
** Never- drinker**	62318 (82.87%)	0.52 (0.47, 0.58)	<0.0001
**Smoking status**			
** Current-smoker**	11788 (15.67%)	ref	
** Ex-smoker**	2918 (3.88%)	0.91 (0.82, 1.01)	0.0724
** Never-smoker**	60498 (80.45%)	0.75 (0.72, 0.79)	<0.0001
**Family history of diabetes**			0.3503
** No**	73750 (98.07%)	ref	
** Yes**	1454 (1.93%)	0.94 (0.82, 1.07)	
**SBP (mmHg)**	118.06 ± 15.90	1.03 (1.03, 1.03)	<0.0001
**DBP (mmHg)**	73.52 ± 10.63	1.03 (1.03, 1.03)	<0.0001
**BMI (kg/m2)**	23.03 ± 3.25	1.12 (1.12, 1.13)	<0.0001
**AST/ALT ratio**	1.24 ± 0.45	0.59 (0.57, 0.62)	<0.0001
**HDL-C (mmol/L)**	1.37 ± 0.29	0.84 (0.79, 0.90)	<0.0001
**TG (mmol/L)**	1.26 ± 0.94	1.19 (1.18, 1.21)	<0.0001
**LDL-C (mmol/L)**	2.69 ± 0.67	1.27 (1.24, 1.31)	<0.0001
**TC (mmol/L)**	4.65 ± 0.89	1.22 (1.19, 1.25)	<0.0001
**BUN (mmol/L)**	4.64 ± 1.19	1.13 (1.11, 1.14)	<0.0001
**SCr (umol/L)**	4.64 ± 1.19	1.13 (1.11, 1.14)	<0.0001
**FPG (mmol/L)**	4.78 ± 0.49	5.30 (5.04, 5.58)	<0.0001

### The results of the association between AST/ALT ratio and prediabetes

**Table 4** showed the Cox proportional hazard regression models, which assessed the exact association between AST/ALT and prediabetes risk. The three adjusted and non-adjusted models were simultaneously presented in **Table 4**. In non-adjusted model, AST/ALT ratio was negatively correlated with prediabetes (HR:0.59, 95% confidence interval (CI): 0.57-0.62, P<0.0001). In the minimally-adjusted model (Model I), after adjusting for gender, age, DBP, SBP, BMI, drinking status, smoking status, and family history of diabetes, no significant change was found in the results (HR: 0.76, 95%CI: 0.72-0.81, P<0.0001). In the fully-adjusted model (Model II), after adjusting for gender, age, DBP, SBP, BMI, HDL-C, TG, LDL-C, TC, Scr, FPG, BUN, drinking status, smoking status and family history of diabetes, AST/ALT ratio was still negatively correlated with prediabetes risk (HR:0.79, 95%CI: 0.75-0.84 P<0.0001). This study demonstrated that an increase in each unit of AST/ALT ratio reduced prediabetes risk by 21%.

**Table 4:** Relationship between AST/ALT ratio and the incident prediabetes in different models.

Variable	Non-adjusted model (HR.,95% CI, P)	Minimally-adjusted model (HR,95% CI, P)	Fully-adjusted model (HR,95% CI, P)	GAM(HR,95% CI, P)
**Total** **AST/ALT ratio**	0.59 (0.57, 0.62) <0.0001	0.76 (0.72, 0.81) <0.0001	0.79 (0.75, 0.84) <0.0001	0.79 (0.75, 0.84) <0.0001
**AST/ALT ratio (quartile)**				
** Q1**	ref	ref	ref	ref
** Q2**	0.80 (0.76, 0.84) <0.0001	0.85 (0.81, 0.90) <0.0001	0.86 (0.81, 0.90) <0.0001	0.85 (0.80, 0.90) <0.0001
** Q3**	0.68 (0.64, 0.72) <0.0001	0.82 (0.77, 0.87) <0.0001	0.84 (0.79, 0.90) <0.0001	0.84 (0.79, 0.89) <0.0001
** Q4**	0.55 (0.52, 0.59) <0.0001	0.75 (0.70, 0.80) <0.0001	0.78 (0.72, 0.83) <0.0001	0.78 (0.73, 0.84) <0.0001
**P for trend**	<0.0001	<0.0001	<0.0001	<0.0001

Crude model: we did not adjust for other covariants.

Minimally-adjusted model: we adjusted for gender, age, family history of diabetes, drinking status, smoking status, SBP, DBP, BMI.

Fully-adjusted model: we adjusted for gender, age, family history of diabetes, drinking status, smoking status, SBP, DBP, BMI, TC, TG, HDL-C, LDL-C, SCr, BUN, FPG.

GAM: All covariates listed in **Table 1** were adjusted. However, continuous covariates were adjusted as nonlinearity.

HR, hazard ratios; CI, confidence interval; Ref, reference; GAM, generalized additive mode; AST/ALT ratio, aspartate aminotransferase to alanine aminotransferase ratio.

### Sensitivity analyses

To examine the robustness of our conclusions, we employed a series of sensitivity analyses. The AST/ALT ratio was transformed into a categorical variable (based on quartile) and reinserted into the models. When the AST/ALT ratio was transformed into a categorical variable, p for the trends were not equal, suggesting a possible nonlinear association between the AST/ALT ratios and prediabetes risk. Additionally, the continuity covariate was inserted into the equation by a GAM. The findings of the GAM model were consistent with the findings of the fully-adjusted model (HR:0.79, 95%CI:0.75-0.84) (**Table 4**). Besides, to evaluate the impact of potential unmeasured confounding variables between the AST/ALT ratio and the risk of prediabetes, this study further generated E-values. The E value for this study was 1.85. Compared to the relative risk and AST/ALT ratio for unmeasured confounding variables, the E value for this study was larger. The results indicated that unknown or unmeasured confounding variables had little effect on the association between the AST/ALT ratio and the risk of prediabetes.

In addition, individuals with a BMI≥24kg/m^2^ were excluded from other sensitivity analyses. After adjusting the confounding factors, there was also a negative association between the AST/ALT ratio and prediabetes risk (HR:0.79, 95%CI: 0.73-0.85) (**Table 5**). Individuals with age≥60 years were also excluded from other sensitivity analyses. The results suggested that after adjusting gender, age, DBP, SBP, BMI, HDL-C, TG, LDL-C, TC, Scr, FPG, BUN, drinking status, smoking status and family history of diabetes, AST/ALT ratio was negatively correlated with prediabetes risk (HR:0.78, 95%CI: 0.73-0.84) (**Table 5**). Furthermore, we excluded drinker (current-drinker and ex-drinker) for sensitivity analyses. The results suggested that AST/ALT ratio was still negatively associated with incident prediabetes in the fully adjusted model (HR:0.80, 95%CI: 0.75-0.85) (**Table 5**). Based on the sensitivity analyses, our findings appeared to be well-robust.

**Table 5:** Relationship between AST/ALT ratio and prediabetes in different sensitivity analyses.

Exposure	Model I (HR,95%CI, P)	Model II (HR,95%CI, P)	Model III (HR,95%CI, P)
**AST/ALT ratio**	0.79 (0.73, 0.85) <0.0001	0.78 (0.73, 0.84) <0.0001	0.80 (0.75, 0.85) <0.0001
**AST/ALT ratio (Quintile)**			
** Q1**	Ref	Ref	Ref
** Q2**	0.87 (0.80, 0.95) 0.0029	0.86 (0.81, 0.91) <0.0001	0.87 (0.81, 0.92) <0.0001
** Q3**	0.82 (0.74, 0.89) <0.0001	0.85 (0.79, 0.91) <0.0001	0.85 (0.79, 0.91) <0.0001
** Q4**	0.74 (0.67, 0.81) <0.0001	0.77 (0.71, 0.84) <0.0001	0.77 (0.71, 0.83) <0.0001
**P for trend**	<0.0001	<0.0001	<0.0001

Model I was sensitivity analysis in participants without BMI≥24kg/m^2^. We adjusted gender, age, family history of diabetes, drinking status, smoking status, SBP, DBP, BMI, TC, TG, HDL-C, LDL-C, SCr, BUN, FPG.

Model II was sensitivity analysis in participants without age≥60 years. We adjusted gender, age, family history of diabetes, drinking status, smoking status, SBP, DBP, BMI, TC, TG, HDL-C, LDL-C, SCr, BUN, FPG.

Model III was sensitivity analysis in participants without drinker (current-drinker and ex-drinker). We adjusted gender, age, family history of diabetes, smoking status, SBP, DBP, BMI, TC, TG, HDL-C, LDL-C, SCr, BUN, FPG.

HR, hazard ratios; CI, confidence, Ref: reference; AST/ALT ratio: aspartate aminotransferase to alanine aminotransferase ratio.

### The analysis of the nonlinear relationship

**Figure 3:**
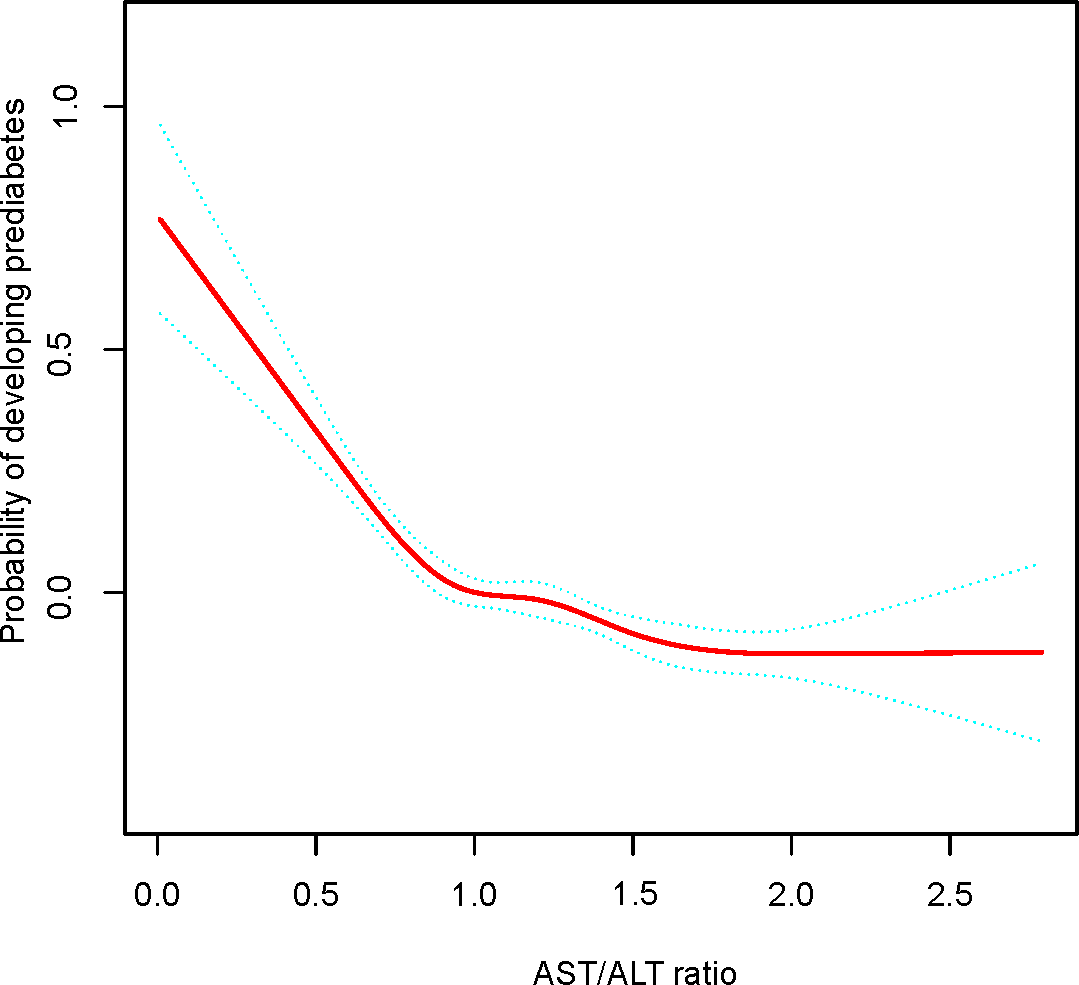
The nonlinear relationship between AST/ALT ratio and incident prediabetes. A nonlinear relationship between them was detected after adjusting for gender, age, family history of diabetes, drinking status, smoking status, SBP, DBP, BMI, TC, TG, HDL-C, LDL-C, SCr, BUN, FPG.

The GAM and the smooth curve fitting (penalty curve method) were applied to verify the nonlinearity in the association between AST/ALT and the risk of prediabetes (**Figure 3**). **Table 6** revealed a nonlinear association between AST/ALT ratio and prediabetes after adjustment for gender, age, DBP, SBP, BMI, HDL-C, TG, LDL-C, TC, Scr, FPG, BUN, drinking status, smoking status, and family history of diabetes. According to a two-piecewise linear regression model, the present study found the inflection point of the AST/ALT ratio was 1.50 (P for log-likelihood ratio test <0.0001). When the AST/ALT ratio was less than 1.50, the AST/ALT ratio was negatively related to the risk of prediabetes (HR:0.70, 95%CI: 0.65-0.76, P<0.0001). In contrast, the relationship tended to be saturated when the AST/ALT ratio was more than 1.50 (HR: 1.01, 95%CI: 0.89-1.15, P=0.8976).

**Table 6:** The result of two-piecewise linear regression model.

Incident prediabetes	HR (95%CI)	P
**Fitting model by standard linear regression**	0.79 (0.75, 0.84)	<0.0001
**Fitting model by two-piecewise linear regression**		
**Inflection point of AST/ALT ratio**	1.50	
** ≤1.50**	0.70 (0.65, 0.76)	<0.0001
** >1.50**	1.01 (0.89, 1.15)	0.8976
**P for log likelihood ratio test**	<0.001	

We adjusted for gender, age, family history of diabetes, drinking status, smoking status, SBP, DBP, BMI, TC, TG, HDL-C, LDL-C, SCr, BUN, FPG.

HR, hazard ratios; CI, confidence; AST/ALT ratio: aspartate aminotransferase to alanine aminotransferase ratio.

### The results of the subgroup analysis

A subgroup analysis was employed in the present study to examine other influencing factors that could have influenced the relationship between AST/ALT ratio and prediabetes risk. Gender, age, DBP, SBP, BMI, smoking status, drinking status, and family history of diabetes were chosen as stratification factors, and the effect size trends of these influencing factors were examined (**Table 7**). In our study, any interactions were examined according to the prior norm. According to the results, the association between AST/ALT ratio and prediabetes risk was not modified by any of the above influencing factors.

**Table 7:** Effect size of AST/ALT ratio on prediabetes in prespecified and exploratory subgroups.

Characteristic	No of participants	HR (95%CI) P value	P for interacion
**Age, years**			0.0605
** <60**	67934	0.82 (0.77, 0.87) <0.0001	
** ≥60**	7270	0.93 (0.83, 1.05) 0.2557	
**Gender**			0.8142
** Male**	41909	0.81 (0.75, 0.87) <0.0001	
** Female**	33295	0.80 (0.73, 0.87) <0.0001	
**BMI (kg/m2)**			0.3382
** < 24**	47638	0.74 (0.69, 0.80) <0.0001	
** ≥24**	27566	0.79 (0.72, 0.85) <0.0001	
**SBP (mmHg)**			0.4985
** < 140**	68621	0.80 (0.75, 0.85) <0.0001	
** ≥140**	6583	0.84 (0.73, 0.97) 0.0140	
**DBP (mmHg)**			0.7741
** <90**	69882	0.79 (0.75, 0.84) <0.0001	
** ≥90**	5322	0.81 (0.69, 0.96) 0.0173	
**Smoking status**			0.1505
** Current smoker**	11788	0.84 (0.73, 0.96) 0.0133	
** Ever smoker**	2918	0.61 (0.45, 0.82) 0.0012	
** Never smoker**	60498	0.80 (0.75, 0.85) <0.0001	
**Drinking status**			0.5173
** Current drinker**	1579	0.88 (0.64, 1.20) 0.4163	
** Ever drinker**	11307	0.74 (0.64, 0.86) <0.0001	
** Never drinker**	62318	0.80 (0.75, 0.85) <0.0001	
**Family history of diabetes**			0.3478
** No**	73750	0.79 (0.75, 0.84) <0.0001	
** Yes**	1454	0.64 (0.42, 1.00) 0.0480	

Above model adjusted for gender, age, family history of diabetes, drinking status, smoking status, SBP, DBP, BMI, TC, TG, HDL-C, LDL-C, SCr, BUN, FPG.

In each case, the model is not adjusted for the stratification variable.

## Discussion

The purpose of our retrospective study was to examine the association between the AST/ALT ratio and prediabetes risk in Chinese subjects. This study demonstrated that increased AST/ALT ratios were associated with a reduced risk of prediabetes. Furthermore, a saturation effect curve was discovered as well, and the relationship between different AST/ALT ratios on prediabetes was examined on the left and right sides of the inflection point. An analysis of subgroups found a stable correlation between AST/ALT ratio and prediabetes risk.

In recent years, prediabetes was reported to have an age-standardized incidence rate of prediabetes were 62.6/1000 person-years (73.8/1000 person-years for males and 51.2/1000 person-years for females) in the general population of China. The age-standardized incidence rates of prediabetes in the present study were 41.5/1000 person-years, lower than the reported level. Due to the more detailed data analysis and stricter selection criteria employed in this study, the apparent discrepancy can be explained.

Prediabetes is the pathological stage that must be crossed in order to progress to type 2 diabetes, with an annual conversion rate between 5–10% (4). Dysfunction of the islets and IR plays a significant role in the onset and progression of prediabetes (30, 31). Previous studies evaluated biochemical markers of liver damage using the AST/ALT ratio. Decreased AST/ALT ratio is one of the best surrogates for nonalcoholic steatohepatitis (32). Recent studies have pointed out that the AST/ALT ratio can be used as one of the surrogate indicators of hyperinsulinemia and insulin resistance (13, 33). In addition, studies have shown that with the increase in the AST/ALT ratio, the future risk of cardiovascular disease, metabolic syndrome, and DM will gradually decrease (14, 15, 34). Patients with prediabetes displayed markers of liver function injury, such as elevated ALT and AST levels (35). Among them, decreased AST/ALT can lead to functional impairment of pancreatic β cells, leading to aggravation of IR, forming a vicious circle, and accelerating the progression from prediabetes to type 2 diabetes (13). In a study of 808 Korean adolescents, Lee and Yang et al. (15) found that a positive relationship existed between ALT and metabolic syndrome prevalence, while a negative relationship existed between the AST/ALT ratio and metabolic syndrome prevalence. In a cross-sectional study involving 2,585 Thais participants, Homsanit M et al. (36) found that after adjusting for demographic and biochemical covariates, the ALT/AST ratio≥1 was significantly associated with metabolic syndrome (OR: 1.72, 95% CI: 1.28-2.32 for females, and OR: 2.30, 95% CI: 1.68-3.16 for male). In a retrospective study of 15,464 Japanese participants, Hua Niu et al. (34) found that when the AST/ALT ratio was ≤0.93, the AST/ALT ratio was negatively associated with incident diabetes (HR:0.14, 95%CI:0.02–0.90, P=0.0385) after adjustment for age, sex, BMI, regular exerciser, waist circumference, smoking, alcohol consumption, γ-glutamyl transpeptidase, HDL-C, TC, TG, HBA1C, FBG, SBP, DBP, fatty liver, AST and ALT. In the present study, we found that an increase of each unit of the AST/ALT ratio reduced the risk of prediabetes by 21% (HR:0.79, 95%CI: 0.75-0.84 P<0.0001) after adjusting for gender, age, DBP, SBP, BMI, HDL-C, TG, LDL-C, TC, Scr, FPG, BUN, drinking status, smoking status and family history of diabetes. Meanwhile, the sensitivity analyses demonstrated that this relationship could still be detected among individuals without BMI≥24kg/m^2^, age≥60 years or drinker (current-drinker and ex-drinker). The above-mentioned efforts have confirmed the relationship’s stability between the AST/ALT ratio and prediabetes risk. It was known that the AST/ALT ratio was the first to be found to be correlated with prediabetes in our study, which may provide valuable insight into the primary prevention of diabetes.

Furthermore, to our knowledge, a nonlinear relationship between the AST/ALT ratio and prediabetes risk was first identified by our study. In this study, we employed a two-piecewise linear regression model to identify a nonlinear relationship between the AST/ALT ratio and prediabetes risk. The inflection point was 1.50 after adjusting for confounders (gender, age, DBP, SBP, BMI, HDL-C, TG, LDL-C, TC, Scr, FPG, BUN, drinking status, smoking status, and family history of diabetes.). Results revealed that when the AST/ALT ratio was less than 1.50, a 1-unit increase in AST/ALT ratio levels was correlated to a 30% reduction in adjusted HR of prediabetes risk (HR:0.70, 95%CI: 0.65-0.76, P<0.0001). However, when AST/ALT ratio was>1.50, the risk of prediabetes was directly proportional to AST/ALT, but it was not statistically significant (HR: 1.01, 95%CI: 0.89-1.15, P=0.8976). The reason might be that prediabetes risk was influenced by other covariates at baseline. It could be seen from **Table S1** that compared with the AST/ALT ratio<1.50 group, subjects with the AST/ALT ratio≥1.50 have generally lower BMI, BUN, FPG, TC, TG, LDL-C, SCr, higher HDL-C, and a higher proportion of females. In addition, the above indicators are protective factors for prediabetes (37). Prediabetes risk was only marginally affected by the AST/ALT ratio when the AST/ALT ratio exceeded 1.50 because of the presence of the above protective factors for prediabetes. Conversely, when the AST/ALT ratio was lower than 1.50, the ability of the above indicators to protect against prediabetes was weakened, and the above indicators had less influence on prediabetes; at this time, the AST/ALT ratio had more influence on prediabetes.

Prediabetes and AST/ALT ratios are linked, but the exact mechanism remains unclear. There are currently several explanations for the AST/ALT ratio leading to prediabetes. AST and ALT are also clinically biochemical markers of liver damage due to their release from hepatocytes as a result of cellular damage. Inflammation in the liver has been reported to impair insulin signaling, resulting in an inability to suppress glucose production and ultimately leading to hyperglycemia (38, 39). A big role is played by systemic insulin resistance which induces reduced glucose uptake and free fatty acids beta-oxidation, and increased gluconeogenesis and *de novo* lipogenesis which leads to NAFLD development (40, 41). As well in NAFLD patients, the increased inflammation and oxidative stress lead to an increase in the release of inflammatory cytokines and a worsening of systemic insulin resistance (40, 41). Moreover, NAFLD is also responsible for increased hepatic insulin resistance, which induces compensatory hyperglycemia, which leads to beta cell dysfunction and relative insulin deficiency (40, 41). Furthermore, since insulin is crucial for regulation and metabolism, organs like the liver are particularly impacted in people with systemic insulin resistance (42). Excess glucose is returned to the liver, where it may hinder gluconeogenesis and encourage lipogenesis when insulin-resistant tissue is unable to increase glucose absorption (42–44). As a result of the fat buildup and inflammation that follows, the liver’s structure is distorted and destroyed, raising ALT levels (13). AST, a vital enzyme for aerobic metabolism, is not only found in the liver but also in other tissues such as the heart, skeletal muscle, kidney, and red blood cells. Insulin no longer effectively allows glucose to enter cells to start aerobic metabolism in insulin-resistant tissues, which reduces AST activity (42, 45). In addition, the AST/ALT ratio is a marker for chronic liver disease (NAFLD or HCV disease, etc.) (16, 46). The risk of diabetes and prediabetes is related to some chronic liver diseases (NAFLD or HCV disease, etc.), which are already known risk factors (25, 47–49). Changes in AST/ALT ratio may reflect a marker of the impact of some chronic liver diseases (NAFLD or HCV disease, etc.) on prediabetes.

Several strengths of our study can be found. First, the present study used a large sample size, and participants came from a variety of centers, representing the Chinese population more accurately. Second, we examined the nonlinear relationship by using a GAM and a smooth curve fitting in order to identify the optimal inflection point for the effect of AST/ALT ratios on prediabetes. Third, results were also rigorously adjusted statistically to reduce the influence of confounding factors, and it ensured the reliability of our findings. Fourth, our results were tested for robustness through sensitivity analyses (the AST/ALT ratio transformation, employing a GAM to put the continuity covariate into the equation as a curve, estimating E-values to examine the potential for unmeasured confounders, subgroup analysis, and reassessing the relationship between AST/ALT ratio and prediabetes after excluding participants with BMI≥24kg/m2, age≥60 years or drinker) to ensure their reliability. Fifth, we employed a subgroup analysis to find other risk factors that might affect the relationship between AST/ALT and prediabetes.

The present study is still subject to some potential shortcomings: (1) We may have missed some new cases of prediabetes because of the use of FPG levels for diagnosing prediabetes in the present study. (2) As with all retrospective studies, our study may have had unmeasured or uncontrolled confounding covariates, such as dietary factors, cytolytic activity, NAFLD, viral hepatitis and alcohol abuse, even though we controlled for known potential confounding covariates. However, E values were calculated to quantify the potential impact of unmeasured confounding covariates, and it was found that unmeasured confounding covariates were unlikely to influence the relationship between the AST/ALT ratio and risk of prediabetes. (3) Since this study only involved Chinese participants, our findings may not be generalizable to other ethnicities. (4) AST/ALT ratio may be biased by the concurrent presence of viral hepatitis and or alcohol abuse (which may increase AST levels and thus increase the ratio). (5) The initial study only measured baseline ALT and AST. Some drugs, such as hepatoprotective drugs, may affect ALT and AST levels. Therefore, ALT and AST may change during follow-up. However, the initial study did not measure the changes in ALT and AST over time. In the future, we will consider designing our study to document more confounding factors, including ALT and AST fluctuations, cytolytic activity, NAFLD, viral hepatitis, alcohol abuse, and use of drugs during follow-up. Therefore, we could explore the impact of changes in the AST/ALT ratio on future prediabetes risk through a GAM model.

## Conclusion

This study showed that the AST/ALT ratio was inversely correlated with the prevalence of prediabetes in Chinese adults. When there is a persistent decrease in the AST/ALT ratio, clinical vigilance for the risk of prediabetes is warranted. Furthermore, there is a nonlinear relationship and saturation effect between the AST/ALT ratio and the risk of prediabetes, with an inflection point of 1.50. When the AST/ALT ratio decreases below 1.50, clinical vigilance for the risk of prediabetes should be high. Therefore, clinically, we reflect the magnitude of the risk of prediabetes by focusing on changes in the AST/ALT ratio.

## Data availability statement

The datasets presented in this study can be found in online repositories. The names of the repository/repositories and accession number(s) can be found in the article/**Supplementary Material**.

## Ethics statement

The studies involving human participants were reviewed and approved by Rich Healthcare Group Review Board. The patients/participants provided their written informed consent to participate in this study.

## Author contributions

CC and XHZ conceived and designed the research, drafted the manuscript. JY and YZ did the statistical analysis. XZ and CX took part in the discussion. YW and XDZ revised the manuscript. All authors contributed to the article and approved the submitted version.

## Funding

This study was supported by the Sanming Project of Medicine in Shenzhen (No. SZSM202111010).

## Conflict of interest

The authors declare that the research was conducted in the absence of any commercial or financial relationships that could be construed as a potential conflict of interest.

## Publisher’s note

All claims expressed in this article are solely those of the authors and do not necessarily represent those of their affiliated organizations, or those of the publisher, the editors and the reviewers. Any product that may be evaluated in this article, or claim that may be made by its manufacturer, is not guaranteed or endorsed by the publisher.

## Abbreviations

BMI, Body mass index; SBP, Systolic blood pressure; DBP, Diastolic blood pressure; FPG, Fasting plasma glucose; TC, Total cholesterol; TG, Triglyceride; HDL-C, high-density lipoprotein cholesterol; LDL-C, Low-density lipid cholesterol; BUN, Blood urea nitrogen; Scr, Serum creatinine; ALT, Alanine aminotransferase; AST, Aspartate aminotransferase; AST/ALT ratio, Aspartate aminotransferase to alanine aminotransferase ratio; IR, Insulin resistance; GAM: Generalized additive models; HR, hazard ratios; CI, Confidence intervals; Ref, Reference.

## References

[1] Saeedi P Petersohn I Salpea P Malanda B Karuranga S Unwin N. Global and regional diabetes prevalence estimates for 2019 and projections for 2030 and 2045: Results from the international diabetes federation diabetes atlas, 9(th) edition. Diabetes Res Clin Pract (2019) 157:107843. doi: 10.1016/j.diabres.2019.107843 31518657

[2] American Diabetes Association. Economic costs of diabetes in the U.S. @ in 2017. Diabetes Care (2018) 41(5):917–28. doi: 10.2337/dci18-0007 PMC5911784 29567642

[3] Echouffo-Tcheugui JB Selvin E. Prediabetes and what it means: The epidemiological evidence. Annu Rev Public Health (2021) 42:59–77. doi: 10.1146/annurev-publhealth-090419-102644 33355476 PMC8026645

[4] Tabak AG Herder C Rathmann W Brunner EJ. Kivimaki m: Prediabetes: a high-risk state for diabetes development. LANCET (2012) 379(9833):2279–90. doi: 10.1016/S0140-6736(12)60283-9 PMC3891203 22683128

[5] Nanditha A Ma RC Ramachandran A Snehalatha C Chan JC Chia KS. Zimmet PZ: Diabetes in Asia and the pacific: Implications for the global epidemic. Diabetes Care (2016) 39(3):472–85. doi: 10.2337/dc15-1536 26908931

[6] Chen L Magliano DJ. Zimmet PZ: The worldwide epidemiology of type 2 diabetes mellitus–present and future perspectives. Nat Rev Endocrinol (2011) 8(4):228–36. doi: 10.1038/nrendo.2011.183 22064493

[7] Mathenge N Fan W Wong ND Hirsch C Delaney CJ Amsterdam EA. Gardin JM: Pre-diabetes, diabetes and predictors of incident angina among older women and men in the cardiovascular health study. Diabetes Vasc Dis Res (2020) 17(1):1142017444. doi: 10.1177/1479164119888476 PMC7510359 31778070

[8] Knowler WC Barrett-Connor E Fowler SE Hamman RF Lachin JM Walker EA. Nathan DM: Reduction in the incidence of type 2 diabetes with lifestyle intervention or metformin. N Engl J Med (2002) 346(6):393–403. doi: 10.1056/NEJMoa012512 11832527 PMC1370926

[9] Hazlehurst JM Woods C Marjot T Cobbold JF. Tomlinson JW: Non-alcoholic fatty liver disease and diabetes. METABOLISM (2016) 65(8):1096–108. doi: 10.1016/j.metabol.2016.01.001 PMC4943559 26856933

[10] Ballestri S Zona S Targher G Romagnoli D Baldelli E Nascimbeni F. Nonalcoholic fatty liver disease is associated with an almost twofold increased risk of incident type 2 diabetes and metabolic syndrome. Evidence systematic Rev meta-analysis J Gastroenterol Hepatol (2016) 31(5):936–44. doi: 10.1111/jgh.13264 26667191

[11] Younossi ZM Golabi P de Avila L Paik JM Srishord M Fukui N. The global epidemiology of NAFLD and NASH in patients with type 2 diabetes: A systematic review and meta-analysis. J Hepatol (2019) 71(4):793–801. doi: 10.1016/j.jhep.2019.06.021 31279902

[12] Kawamoto R Kohara K Kusunoki T Tabara Y Abe M Miki T. Alanine aminotransferase/aspartate aminotransferase ratio is the best surrogate marker for insulin resistance in non-obese Japanese adults. Cardiovasc Diabetol (2012) 11:117. doi: 10.1186/1475-2840-11-117 23020992 PMC3499385

[13] Visaria A Pai S Cheung M Ahlawat S. Association between aspartate aminotransferase-to-alanine aminotransferase ratio and insulin resistance among US adults. Eur J Gastroenterol Hepatol (2022) 34(3):316–23. doi: 10.1097/MEG.0000000000002215 34074988

[14] Weng SF Kai J Guha IN Qureshi N. The value of aspartate aminotransferase and alanine aminotransferase in cardiovascular disease risk assessment. Open Heart (2015) 2(1):e272. doi: 10.1136/openhrt-2015-000272 PMC4548065 26322236

[15] Lee K Yang JH. Which liver enzymes are better indicators of metabolic syndrome in adolescents: the fifth Korea national health and nutrition examination survey, 2010. Metab Syndr Relat Disord (2013) 11(4):229–35. doi: 10.1089/met.2012.0153 23451816

[16] Bayrak M. Non-invasive diagnosis of nonalcoholic fatty liver disease: impact of age and other risk factors. Aging MALE (2020) 23(5):1275–82. doi: 10.1080/13685538.2020.1763293 32396414

[17] Chen L Zhang K Li X Wu Y Liu Q Xu L. Association between aspartate aminotransferase to alanine aminotransferase ratio and incidence of type 2 diabetes mellitus in the Japanese population: A secondary analysis of a retrospective cohort study. Diabetes Metab Syndr Obes (2021) 14:4483–95. doi: 10.2147/DMSO.S337416 PMC8590482 34785918

[18] Chen Y Zhang XP Yuan J Cai B Wang XL Wu XL. Association of body mass index and age with incident diabetes in Chinese adults: a population-based cohort study. BMJ Open (2018) 8(9):e21768. doi: 10.1136/bmjopen-2018-021768 PMC6169758 30269064

[19] American Diabetes Association. 2. classification and diagnosis of diabetes: Standards of medical care in diabetes-2018. Diabetes Care (2018) 41(Suppl 1):S13–27. doi: 10.2337/dc21-S002 29222373

[20] Qin H Chen Z Zhang Y Wang L Ouyang P Cheng L. Triglyceride to high-density lipoprotein cholesterol ratio is associated with incident diabetes in men: A retrospective study of Chinese individuals. J Diabetes Investig (2020) 11(1):192–8. doi: 10.1111/jdi.13087 PMC6944823 31145539

[21] White IR Royston P Wood AM. Multiple imputation using chained equations: Issues and guidance for practice. Stat Med (2011) 30(4):377–99. doi: 10.1002/sim.4067 21225900

[22] Vandenbroucke JP von Elm E Altman DG Gotzsche PC Mulrow CD Pocock SJ. Strengthening the reporting of observational studies in epidemiology (STROBE): explanation and elaboration. Int J Surg (2014) 12(12):1500–24. doi: 10.1016/j.ijsu.2014.07.014 25046751

[23] Haneuse S VanderWeele TJ Arterburn D. Using the e-value to assess the potential effect of unmeasured confounding in observational studies. JAMA (2019) 321(6):602–3. doi: 10.1001/jama.2018.21554 30676631

[24] Zhu F Chen C Zhang Y Chen S Huang X Li J. Elevated blood mercury level has a non-linear association with infertility in U.S. women: Data from the NHANES 2013-2016. Reprod Toxicol (2020) 91:53–8. doi: 10.1016/j.reprotox.2019.11.005 31756438

[25] Zheng X Cao C He Y Wang X Wu J Hu H. Association between nonalcoholic fatty liver disease and incident diabetes mellitus among Japanese: a retrospective cohort study using propensity score matching. Lipids Health Dis (2021) 20(1):59. doi: 10.1186/s12944-021-01485-x 34130693 PMC8207755

[26] Mullee A Romaguera D Pearson-Stuttard J Viallon V Stepien M Freisling H. Association between soft drink consumption and mortality in 10 European countries. JAMA Intern Med (2019) 179(11):1479–90. doi: 10.1001/jamainternmed.2019.2478 PMC6724165 31479109

[27] Keidel D Anto JM Basagana X Bono R Burte E Carsin AE. The role of socioeconomic status in the association of lung function and air pollution-a pooled analysis of three adult ESCAPE cohorts. Int J Environ Res Public Health (2019) 16(11):1901. doi: 10.3390/ijerph16111901 PMC6603717 31146441

[28] Robson ME Tung N Conte P Im SA Senkus E Xu B. OlympiAD final overall survival and tolerability results: Olaparib versus chemotherapy treatment of physician's choice in patients with a germline BRCA mutation and HER2-negative metastatic breast cancer. Ann Oncol (2019) 30(4):558–66. doi: 10.1093/annonc/mdz012 PMC6503629 30689707

[29] von Elm E Altman DG Egger M Pocock SJ Gotzsche PC Vandenbroucke JP. The strengthening the reporting of observational studies in epidemiology (STROBE) statement: guidelines for reporting observational studies. Int J Surg (2014) 12(12):1495–9. doi: 10.1016/j.ijsu.2014.07.013 25046131

[30] Faerch K Vistisen D Pacini G Torekov SS Johansen NB Witte DR. Insulin resistance is accompanied by increased fasting glucagon and delayed glucagon suppression in individuals with normal and impaired glucose regulation. DIABETES (2016) 65(11):3473–81. doi: 10.2337/db16-0240 27504013

[31] Wang T Lu J Shi L Chen G Xu M Xu Y. Association of insulin resistance and beta-cell dysfunction with incident diabetes among adults in China: a nationwide, population-based, prospective cohort study. Lancet Diabetes Endocrinol (2020) 8(2):115–24. doi: 10.1016/S2213-8587(19)30425-5 31879247

[32] Zou Y Zhong L Hu C Sheng G. Association between the alanine aminotransferase/aspartate aminotransferase ratio and new-onset non-alcoholic fatty liver disease in a nonobese Chinese population: a population-based longitudinal study. Lipids Health Dis (2020) 19(1):245. doi: 10.1186/s12944-020-01419-z 33239040 PMC7690093

[33] Marchesini G Brizi M Bianchi G Tomassetti S Bugianesi E Lenzi M. Nonalcoholic fatty liver disease: a feature of the metabolic syndrome. Diabetes (2001) 50(8):1844–50. doi: 10.2337/diabetes.50.8.1844 11473047

[34] Niu H Zhou Y. Nonlinear relationship between AST-to-ALT ratio and the incidence of type 2 diabetes mellitus: A follow-up study. Int J Gen Med (2021) 14:8373–82. doi: 10.2147/IJGM.S341790 PMC8608244 34819745

[35] Qin G Lu L Xiao Y Zhu Y Pan W Xu X. A cross-sectional study of the relationship between serum liver enzymes level and the incidence of impaired fasting glucose in males and females. Med Sci Monit (2014) 20:1319–25. doi: 10.12659/MSM.890698 PMC4136962 25066107

[36] Homsanit M Sanguankeo A Upala S Udol K. Abnormal liver enzymes in Thai patients with metabolic syndromes. J Med Assoc Thai (2012) 95(3):444–51. 22550846

[37] Gong R Liu Y Luo G Liu W Jin Z Xu Z. Associations of TG/HDL ratio with the risk of prediabetes and diabetes in Chinese adults: A Chinese population cohort study based on open data. Int J Endocrinol (2021) 2021:9949579. doi: 10.1155/2021/9949579 34306073 PMC8282372

[38] Yadav D Choi E Ahn SV Baik SK Cho YZ Koh SB. Incremental predictive value of serum AST-to-ALT ratio for incident metabolic syndrome: The ARIRANG study. PloS One (2016) 11(8):e161304. doi: 10.1371/journal.pone.0161304 PMC4999188 27560931

[39] Hsueh WA Quinones MJ. Role of endothelial dysfunction in insulin resistance. Am J Cardiol (2003) 92(4A):10J–7J. doi: 10.1016/S0002-9149(03)00611-8 12957322

[40] Rinaldi L Pafundi PC Galiero R Caturano A Morone MV Silvestri C. Mechanisms of non-alcoholic fatty liver disease in the metabolic syndrome. a narrative review. Antioxidants (Basel) (2021) 10(2):270. doi: 10.3390/antiox10020270 33578702 PMC7916383

[41] Acierno C Caturano A Pafundi PC Nevola R Adinolfi LE Sasso FC. Nonalcoholic fatty liver disease and type 2 diabetes: pathophysiological mechanisms shared between the two faces of the same coin. Explor Med (2020) 1(5):287–306. doi: 10.37349/emed.2020.00019

[42] Abel ED O'Shea KM Ramasamy R. Insulin resistance: metabolic mechanisms and consequences in the heart. Arterioscler Thromb Vasc Biol (2012) 32(9):2068–76. doi: 10.1161/ATVBAHA.111.241984 PMC3646067 22895668

[43] Kahn BB Flier JS. Obesity and insulin resistance. J Clin Invest (2000) 106(4):473–81. doi: 10.1172/JCI10842 PMC380258 10953022

[44] Han HS Kang G Kim JS Choi BH Koo SH. Regulation of glucose metabolism from a liver-centric perspective. Exp Mol Med (2016) 48:e218. doi: 10.1038/emm.2015.122 26964834 PMC4892876

[45] Swarup S Goyal A Grigorova Y Zeltser R. Metabolic syndrome.StatPearls [Internet]. Physiol Rev (2020) 98(4):2133–223. doi: 10.1152/physrev.00063.2017

[46] Treem WR Palmer M Lonjon-Domanec I Seekins D Dimick-Santos L Avigan MI. Consensus guidelines: Best practices for detection, assessment and management of suspected acute drug-induced liver injury during clinical trials in adults with chronic viral hepatitis and adults with cirrhosis secondary to hepatitis b, c and nonalcoholic steatohepatitis. Drug Saf (2021) 44(2):133–65. doi: 10.1007/s40264-020-01014-2 PMC7847464 33141341

[47] Fabiani S Fallahi P Ferrari SM Miccoli M Antonelli A. Hepatitis c virus infection and development of type 2 diabetes mellitus: Systematic review and meta-analysis of the literature. Rev Endocr Metab Disord (2018) 19(4):405–20. doi: 10.1007/s11154-017-9440-1 29322398

[48] Galiero R Caturano A Vetrano E Cesaro A Rinaldi L Salvatore T. Pathophysiological mechanisms and clinical evidence of relationship between nonalcoholic fatty liver disease (NAFLD) and cardiovascular disease. Rev Cardiovasc Med (2021) 22(3):755–68. doi: 10.31083/j.rcm2203082 34565074

[49] Sasso FC Pafundi PC Caturano A Galiero R Vetrano E Nevola R. Impact of direct acting antivirals (DAAs) on cardiovascular events in HCV cohort with pre-diabetes. Nutr Metab Cardiovasc Dis (2021) 31(8):2345–53. doi: 10.1016/j.numecd.2021.04.016 34053830

