# Supplementary material for: Nonlinear relationship between aspartate aminotransferase to alanine aminotransferase ratio and the risk of prediabetes: A retrospective study based on chinese adults
Source: Front Endocrinol (Lausanne). 2022 Oct 25;13:1041616. doi: 10.3389/fendo.2022.1041616 (PMC9640919; doi:10.3389/fendo.2022.1041616)
Supplement: Supplementary file 2 [file Table_1.pdf]

Variable	AST/ALT ratio≤1.5	AST/ALT ratio >1.5	P-value
Participants	55422	19782	
Gender			<0.001
Male	36256 (65.42%)	5653 (28.58%)	
Female	19166 (34.58%)	14129 (71.42%)	
Age(years)	40.91 ± 11.59	40.87 ± 13.38	0.679
Drinking status			
Current-smoker	1287 (2.32%)	292 (1.48%)	
Ex-smoker	9652 (17.42%)	1655 (8.37%)	
Never-smoker	44483 (80.26%)	17835 (90.16%)	
Smoking status			<0.001
Current-smoker	10250 (18.49%)	1538 (7.77%)	
Ex-smoker	2550 (4.60%)	368 (1.86%)	
Never-smoker	42622 (76.90%)	17876 (90.36%)	
Family history of diabetes			0.974
No	54351 (98.07%)	19399 (98.06%)	
Yes	1071 (1.93%)	383 (1.94%)	
SBP (mmHg)	119.43 ± 15.65	114.22 ± 15.96	<0.001
DBP (mmHg)	74.47 ± 10.67	70.87 ± 10.05	<0.001
BMI (kg/m2)	23.63 ± 3.24	21.37 ± 2.65	<0.001
HDL-C (mmol/L)	1.34 ± 0.29	1.44 ± 0.29	<0.001
TG (mmol/L)	1.38 ± 1.00	0.95 ± 0.61	<0.001
LDL-C (mmol/L)	2.72 ± 0.67	2.59 ± 0.65	
TC (mmol/L)	4.69 ± 0.89	4.54 ± 0.87	<0.001
BUN (mmol/L)	4.72 ± 1.18	4.41 ± 1.18	<0.001
SCr (umol/L)	72.72 ± 15.51	65.56 ± 15.93	
FPG (mmol/L)	4.80 ± 0.48	4.73 ± 0.51	<0.001
